# SIRT1 Prevents Lens Epithelial Cell Senescence During Age‐Related Cataract via Regulating p66Shc

**DOI:** 10.1111/acel.70155

**Published:** 2025-06-26

**Authors:** Huirui Liu, Liyao Sun, Yu Mi, Yi Gao, Jialin Luo, Fengchun Kang, Yujing Bai, Xiaohan Yu, Hongyan Ge

**Affiliations:** ^1^ Eye Hospital The First Affiliated Hospital of Harbin Medical University Harbin China; ^2^ NHC Key Laboratory of Cell Transplantation Heilongjiang China; ^3^ Southwest Eye Hospital Southwest Hospital, Third Military Medical University Chongqing China

**Keywords:** cellular senescence, lens epithelial cell, nicotinamide mononucleotide, p66Shc, SIRT1

## Abstract

Lens epithelial cell (LEC) senescence is one of the key pathological processes of age‐related cataract (ARC) and is associated with oxidative stress, mitochondrial dysfunction, and protein aggregation. This study aimed to elucidate the pathogenesis of LEC senescence in ARC. The protein expression level of silencing regulatory protein 1 (SIRT1) and aptamer protein (p66Shc) was quantified. Reactive oxygen species (ROS) and mitochondrial superoxide levels were measured to evaluate cellular oxidative stress. Senescence‐associated protein expression (p21 and p53) and SA‐β‐galactosidase staining were employed to assess the aging status of LEC. Targeted metabolic analysis was conducted to explore energy changes during LEC senescence, and mitochondrial morphology and function were assessed in the cell models. The aging and damage conditions of the lens in ARC rats were evaluated through histological staining, transmission electron microscopy, expression of senescence‐related proteins, and oxidative stress markers. We comprehensively investigated the downregulation of SIRT1 expression and the upregulation of p66Shc expression in human cataract samples, UVB‐induced rat cataract models, and UVB‐treated LEC. SIRT1 could alleviate UVB‐induced oxidative stress, as well as mitochondrial dysfunction, inhibiting p66Shc expression in LEC. Nicotinamide mononucleotide (NMN) effectively alleviated the abnormal expression of aging‐related proteins and inhibited mitochondrial morphological and functional disorders by activating SIRT1. In conclusion, NMN activated SIRT1, inhibiting mitochondrial dysfunction, oxidative stress, and senescence in LEC, delaying lens opacity. This mechanism could be associated with the onset and progression of ARC, providing a new strategy for its prevention and treatment.

## Graphical abstract

Schematic illustration of NMN‐mediated alleviation of age‐related cataract via SIRT1/p66Shc pathway–dependent inhibition of mitochondrial dysfunction and LEC senescence.

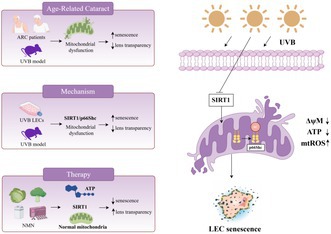

**Abbreviations**
ARC: age‐related cataract
ATP: adenosine triphosphate
Co‐IP: co‐immunoprecipitation
HE: hematoxylin and eosin
IF: Immunofluorescence
LEC: lens epithelial cell
MMP: mitochondrial membrane potential
mtROS: mitochondrial reactive oxygen species
NAD+: nicotinamide adenine dinucleotide
NMN: nicotinamide mononucleotide
qPCR: real time polymerase chain reaction
ROS: reactive oxygen species
SA‐β‐gal: senescence β‐galactosidase
SD: Sprague–Dawley
SEMs: standard errors
siRNAs: small interfering RNAs
TEM: transmission electron microscopy
UV: ultravolet radiation
WB: western blot

## 1 Introduction

Age‐related cataract (ARC) is a leading cause of blindness worldwide (Cicinelli et al. 2022), with approximately 187.84 million individuals aged 45–89 years affected in China by 2050 (Song et al. 2018). Clinically, ARC is morphologically classified into three types, with the cortical type accounting for nearly 28.6% and being the predominant form in China (Tang et al. 2016; Zhang et al. 2011). The inevitable risks of surgical complications and high costs make surgical intervention, although effective, a less desirable treatment option for ARCs (Cooksley et al. 2024; Rao et al. 2011), highlighting the urgent need for pharmacological intervention strategies. This, in turn, drives ongoing research into the pathogenesis of ARC.

Cellular senescence, a stable arrest of the cell cycle, has emerged as a key contributor to the early pathological processes of age‐related diseases (Gurkar et al. 2025). It is characterized by the upregulation of cell cycle inhibitors (such as p21 and p53), increased SA‐β‐galactosidase (SA‐β‐gal) activity, mitochondrial dysfunction, and elevated reactive oxygen species (ROS) levels (Teng et al. 2022; Qin et al. 2025; Soleimani et al. 2023). Cellular senescence represents a plastic form of cell fate, which, unlike cell death, retains metabolic activity and is potentially reversible through molecular or pharmacological interventions (Pan et al. 2022; Nadeem et al. 2025). Nevertheless, the potential role of cell senescence as a mechanism of lens epithelial cell (LEC) during ARC requires further investigation. ARC is the result of multifactorial influences, initiated by various intrinsic and extrinsic triggers, including aging processes, ultraviolet B (UVB) radiation, tobacco use, and alcohol intake (Hong et al. 2022). A systematic analysis of existing epidemiological investigated that cumulative UVB exposure significantly increases the risk of age‐related cortical cataract (Li et al. 2021; McCarty et al. 2000). Despite different pathogenic triggers, they all converge on a common molecular mechanism: the induction of oxidative stress in LEC (Braakhuis et al. 2019; Smith et al. 2016; Huynh et al. 2021).

SIRT1 has been shown to play important roles in the regulation of oxidative stress, mitochondrial function and aging‐related diseases (Li, et al. 2023; Jiang et al. 2024). Notably, studies have shown that decreased SIRT1 activity can lead to cataracts in SD rats under UVB irradiation (Wu et al. 2023; Hua et al. 2019). p66Shc has been reported to be involved in a variety of processes related to aging and metabolic diseases (Pei et al. 2019; Zhang et al. 2022; Potes et al. 2024). In the aging retinal epithelium caused by ROS accumulation, the SIRT1/p66Shc pathway plays a key role in age‐related susceptibility to oxidative stress (Tong et al. 2019). However, the underlying regulating mechanism of SIRT1/p66Shc in ARC is still not clear.

Nicotinamide mononucleotide (NMN) has been shown to directly promote the synthesis of NAD+ (Nadeeshani et al. 2021), effectively alleviating age‐related physiological decline in mouse models without causing obvious deleterious symptoms (Mills et al. 2016). Studies have shown that NAD+ levels decrease with normal aging (Rajman et al. 2018) and that increasing NAD+ can extend the lifespan of a variety of model organisms, including yeast, fruit flies, and nematodes (Balan et al. 2008; Mouchiroud et al. 2013). The latest research indicates that the regeneration of senescent cells may be re‐adjusted to the disrupted metabolic and transcriptional states through the pathways of new synthesis and recycling (NMN‐NAD+) (Chen and Skutella 2022). In addition, NAD+ acts as a cosubstrate for a variety of enzymes (such as SIRT1 and PARP) and participates in the regulation of important biological processes, such as genome stability and gene transcription (Navas and Carnero 2021). To assess the therapeutic potential of targeting LEC senescence in ARC, we treated UVB‐exposed rats with the senomorphic NAD+ precursor, NMN.

In this study, the UVB‐induced ARC model was used to investigate the effect of NMN on LEC senescence, revealing the important role of the SIRT1/p66Shc pathway. SIRT1 inhibited UVB‐induced mitochondrial dysfunction and LEC senescence. Inhibition of SIRT1 leads to an increase in p66Shc expression, which in turn exacerbates oxidative stress and cellular senescence caused by UVB. Metabolomic analysis revealed the pivotal role of energy metabolites, particularly ATP, in UVB‐induced cellular responses. In summary, UVB exposure reduces SIRT1 expression, leading to the upregulation of p66Shc, which in turn promotes oxidative stress, accelerates LEC senescence, and contributes to lens opacity. These findings provide insights into ARC prevention and treatment strategies.

## 2 Materials and Methods

### 2.1 Human Samples

This study was approved by the First Affiliated Hospital of Harbin Medical University Ethics Committee in accordance with the Declaration of Helsinki. Owing to ethical constraints and the impracticality of obtaining anterior lens capsules from healthy individuals, age‐matched normal human lens samples are difficult to acquire for experimental comparison. Before the operation, the degree of lens opacity in cataract patients was evaluated using the modified Lens Opacities Classification System III (LOCS III) (Bencić et al. 2005). Quantitative scores were given for cortical opacity (C), nuclear opacity (NO/NC), and posterior subcapsular opacity (P) based on the standard chart to determine the type and degree of cataract. During the operation, the Emery‐Little nuclear hardness grading standard (Emery grading system) was used to assess the texture of the lens nucleus and the surgical experience, and the samples were classified into different hardness grades (American Academy of Ophthalmology 2015). This system classified the lens nucleus based on its color and hardness into grades II and V, and this grading was subjectively evaluated by the surgeon during the operation based on the resistance of the nucleus removal and the ultrasonic energy demand. Although the assessment methods were different, the common purpose of both was to clearly determine the severity and characteristics of cataracts, thereby providing reference for sample grouping, surgical strategy formulation, and experimental research. All patients provided written informed consent for the use of their samples. The anterior lens capsules were obtained by the same experienced surgeon during capsulorrhexis at the First Affiliated Hospital of Harbin Medical University. The tissue samples were immediately frozen in liquid nitrogen and stored at −80°C. All patient information is shown in Table 1 and Table S1.

**TABLE 1:** Study population characteristics of the participants in this study (mean ± SD).

Characteristic	Grade II (*n* = 32)	Grade V (*n* = 35)	*p*
Age (years)	62.06 ± 9.4	63.86 ± 8.96	0.427^a^
Gender (male/female)	7/9	19/16	

^a^ Student's *t*‐test.

### 2.2 Experimental Animals

Six‐week‐old male Sprague–Dawley (SD) rats were purchased from the Experimental Animal Center of the Second Affiliated Hospital of Harbin Medical University. SD rats were housed in the Department of Experimental Animals of Harbin Medical University. All animal experiments were performed in accordance with the ARVO statement for the Use of Animals in Ophthalmic and Vision Research and the Animal Ethics Committee of the First Affiliated Hospital of Harbin Medical University. All rats were anesthetized via intraperitoneal injection of a mixture of 90 mg/kg ketamine and 15 mg/kg xylazine. Tropicamide epinephrine was then instilled into both eyes. Next, only one eye (right eye) was exposed once for 30 min under a handheld UV lamp (LUYOR LEB‐280 L, USA) at 312 nm and 8 W delivering a dose of 0.9 J/cm^2^, as measured by UVB sensor (TM‐213, Tenmars, China). Irradiation was performed once per day for seven consecutive days. The rats in the NMN group were pretreated 2 days before the experiment via intraperitoneal injection of NMN (500 mg/kg, HY‐F0004; MCE, USA), followed by subconjunctival injections of 50 μL of NMN once per day for nine consecutive days. The rats were killed by CO_2_ asphyxiation, the lens was immediately removed through the posterior approach under sterile conditions, and the lens was completely separated along the lens edge. The lens was photographed through a stereoscope.

### 2.3 Cell Culture and Treatment

The human lens epithelial cell line B‐3 (HLE‐B3) was purchased from the American Type Culture Collection (ATCC Cat# CRL‐11421, RRID: CVCL_6367) and cultured in a humidified incubator at 37°C with 5% CO_2_ in modified Eagle's medium supplemented with 15% fetal bovine serum and 1% penicillin–streptomycin. HLE‐B3 cells were always maintained in 15% fetal bovine serum. HLE‐B3 cells were completely covered with cold PBS and were exposed to UVB light with a total output power of 8 W for less than 200 s. The cells were pretreated with NMN (0.5 mM, HY‐F0004; MCE, USA) for 2 h in the dark and then incubated in a culture medium with or without UVB irradiation for 24 h. HLE‐B3 cells were pretreated with SIRT1 inhibitor Ex‐527 (10 μM, HY‐15452; MCE, USA) for 2 h for SIRT1 inhibition experiments.

### 2.4 siRNA and Plasmid Transfection

LECs were seeded in six‐well plates and transfected with small interfering RNAs (siRNAs), including negative control (siNC), SIRT1 (siSIRT1), and p66Shc (sip66Shc), purchased from RiboBio. In knockdown experiments, LEC were transfected with 36 μmol/L siRNA and Lipofectamine 2000 (Invitrogen). The sequences of siRNAs were as follows: human SIRT1 siRNA (5′‐GTATTGCTGAACAGATGGAA‐3′); human p66Shc siRNA (5′‐CCACUACCCUGUGCUCCUUTT‐3′) and negative control (NC) siRNA (5′‐UUCUCCGAACGUGUCACGU‐3′). siSIRT1 was first transfected into the cells, and then sip66Shc was transfected into the cells 24 h later.

The pcDNA3.1‐SIRT1 (oeSIRT1), pcDNA3.1‐p66Shc (oep66Shc), and control (oeNC) plasmid were purchased from Jima Bio. Plasmid transfection was performed by jetPRIME (Polyplus), with a DNA‐to‐jetPRIME ratio of 1:3 (w/v). According to the manufacturer's protocol, when LEC reached 70% confluence, the transfection mixture was fully premixed for 20 min and then added to the culture medium. 24 h after transfection, the transfected cells were used for subsequent experiments.

### 2.5 Senescence β‐Galactosidase Staining (SA‐β‐gal)

SA‐β‐gal, a senescence biomarker, was detected using a senescent cell histochemical staining kit (C0602; Beyotime, China) according to the manufacturer's protocol. Positively stained senescent LECs appeared blue under a light microscope (Vert.A1, Zeiss, Germany).

### 2.6 Real Time Polymerase Chain Reaction (qPCR)

Total RNA was extracted from treated cells using a simple total RNA extraction kit (BSC52M1; BioFlux, USA). Total RNA was reverse transcribed into cDNA. Relative RNA expression was detected using QPCR SYBR Green Master Mix (G3326; Servicebio, China). Relative mRNA levels were calculated using the 2^−ΔΔCt^ method. The primer sequences are shown in Table S2. GAPDH was used as a housekeeping gene.

### 2.7 Western Blot Analysis (WB)

In brief, protein was extracted from cells and anterior lens capsules using radioimmunoprecipitation assay buffer (Beyotime Biotechnology, China) containing 1 mM PMSF. After vortexing, the cells or tissues were centrifuged at 12,000 rpm for 15 min. After sodium dodecyl sulfate–polyacrylamide gel electrophoresis, the proteins were transferred to polyvinylidene fluoride membranes, which were blocked with 5% skim milk at room temperature for 1 h and then incubated with primary antibodies at 4°C overnight. The primary antibodies are shown in Table S3. The membrane was washed three times with TBST and incubated with horseradish peroxidase‐conjugated secondary antibody (1:5000) for 1 h. The protein bands were scanned using a machine. The quantitative statistics of the protein bands were estimated using Image J software.

### 2.8 Hematoxylin and Eosin Staining (HE)

The lenses of the rats were fixed with Davidson's fixative solution, dehydrated and then paraffin embedded in paraffin. First, hematoxylin solution (G1120; Solarbio, China) was used to visualize nuclei. The samples were then rinsed with running tap water, and then, the cytoplasm of the cells was stained with eosin solution. Finally, images were captured with a microscope.

### 2.9 Immunofluorescence Assay (IF)

The anterior capsule and cells of treated rat lenses were collected, fixed, permeabilized, and blocked. Subsequently, they were incubated with primary antibodies (1:100, p21, p53) overnight at 4°C and with secondary antibodies (1:100 goat anti‐rabbit [ZF‐0316, ZSGB‐BIO] and anti‐mouse [ZF‐0313, ZSGB‐BIO]) for 1 h at room temperature and then counterstained with DAPI. Finally, IF was assessed by acquiring images with a Zeiss fluorescence microscope system (Vert.A1, Zeiss, Germany) and analyzing the results using Image J software (version 2.1.0/1.53c).

### 2.10 ROS Measurement

The level of mitochondrial reactive oxygen species (mtROS) was measured using MitoSOX Red (M36007; Thermo Fisher Scientific, USA). The cells were incubated with 500 nM MitoSOX Red for 30 min at 37°C and then imaged using a fluorescence microscope (Vert.A1, Zeiss, Germany). The level of ROS in cells was measured using DHE (HY‐D0079, MCE, USA). The cells were incubated with 5 μM DHE for 30 min at 37°C and then imaged using a fluorescence microscope (Vert.A1, Zeiss, Germany). The results were analyzed using Image J (version 2.1.0/1.53c).

### 2.11 Mitochondrial Membrane Potential (MMP)

To assess the MMP, JC‐1 (C2003S; Beyotime, China) was used. JC‐1 probe was prepared according to the manufacturer's protocol and added to cells, followed by incubation at 37°C for 40 min. Imaging was performed using a fluorescence microscope (IX73, Olympus, Japan). The results were analyzed using Image J (version 2.1.0/1.53c).

### 2.12 ATP Measurement

ATP production was assessed using a kit according to the manufacturer's instructions (HY‐D0079; Beyotime, China). Briefly, cells were lysed and centrifuged at 12,000 g for 5 min at 4°C. The resulting supernatant was added to a black 96‐well plate containing ATP assay working solution. Chemiluminescence was detected using a SpectraMax MiniReader (Molecular Devices, USA).

### 2.13 NAD+/NADH Measurement

NAD+ levels in the supernatant were measured according to the manufacturer's instructions (S0175; Beyotime, China). Cell supernatants were aliquoted and divided into two aliquots, one for the measurement of total NAD (NAD_total_). For NADH, samples were heated to 60°C for 30 min to decompose NAD+ according to the manufacturer's instructions. The optical density at 450 nm was read using a hybrid reader (SpectraMax Mini Reader, Molecular Devices, USA). NAD+ was calculated using the formula NAD+ = NAD_total_ − NADH.

### 2.14 SIRT1 Enzyme Activity Assay

To measure SIRT1 enzyme activity, cells were seeded in 10 cm dishes. First, the cells were lysed in ice‐cold lysis buffer, and after sonication and incubation on ice for 30 min, the lysates were centrifuged at 12,000 rpm for 15 min, and the supernatant was collected for subsequent assays. Next, the protein concentration was determined using a BCA assay, and the enzyme activity was determined using a SIRT1 activity assay kit (ab156065; Abcam, USA) with equal amounts of cell lysates. SIRT1 enzyme activity was quantitatively assessed by the fluorescence signal emitted by the fluorescent substrate peptide at Ex/Em = 350/460 nm using a microplate reader.

### 2.15 Metabolite Analysis

Based on the AB Sciex QTRAP 6500 LC–MS/MS platform, MetWare (http://www.metware.cn/) can identify each metabolite.

### 2.16 Molecular Docking

The structures of drugs and key target proteins were obtained from the PubChem database (https://pubchem.ncbi.nlm.nih.gov) and the PDB database (https://www.rcsb.org). Protein structures were dehydrated and liganded using PyMol software and saved, and molecular docking was performed using AutoDock Vina software. Visualization was performed using PyMol software.

### 2.17 Transmission Electron Microscopy (TEM)

The anterior lens capsule and cells were fixed with 2.5% glutaraldehyde. The tissue and cells were then washed with PBS and fixed with 1% osmium (VIII) oxide for 1 h. After dehydration with ethanol, the samples were embedded in epoxy resin. After processing, TEM was performed. Then, images were taken by a histologist with experience in TEM using a transmission electron microscope (H‐7700, Hitachi High‐Technologies, Tokyo, Japan) at an accelerating voltage of 100 kV.

### 2.18 Co‐Immunoprecipitation (Co‐IP)

Briefly, cells were lysed in IP lysis buffer (P0013; Beyotime, China). The cell lysates were incubated with primary antibodies overnight at 4°C, and the targeted immune complexes were captured with protein A/G Magnetic Beads (HY‐K0202, MCE, USA), and eluted with elution buffer. The antibodies used for IP were as follows: anti‐SIRT1 (8469s, Cell Signaling Technology), anti‐p66Shc (AF6245, Affinity), and anti‐IgG (P2267, Beyotime). The immunoprecipitates were subjected to SDS‐PAGE as described above.

### 2.19 Markers of Oxidative Stress Measurement

The lenses of rats were isolated from the rat eyeballs. Following the manufacturer's instructions (bc0025; Solarbio, China), the samples were reacted with thiobarbituric acid, and then, the level of MDA was determined via colorimetric analysis (532, 600 nm). To determine the antioxidant levels in LEC after UVB, GSH (S0053; Beyotime, China) activity was measured. GSH activity was determined by the reaction of GSH with dithionitrobenzene. The absorbance at 412 nm was measured to determine GSH activity.

### 2.20 Statistical Analysis

All experiments were independently repeated at least three times, and the data are reported as means ± standard errors (SEMs). GraphPad Prism 9 software was used for statistical analyses. For data with a normal distribution, Student's *t‐*test was performed to analyzing data between two groups, one‐way analysis of variance was performed for analyzing data among multiple groups, and Tukey's test was performed for comparisons among multiple groups. The Kruskal–Wallis test was used to compare nonnormally distributed data. *p* values of less than 0.05 were considered statistically significant (*p < 0.05, **p < 0.01, ***p < 0.001, ****p < 0.0001).

## 3 RESUITS

### 3.1 SIRT1 Was Downregulated in UVB‐Induced LEC Senescence

**FIGURE 1:**
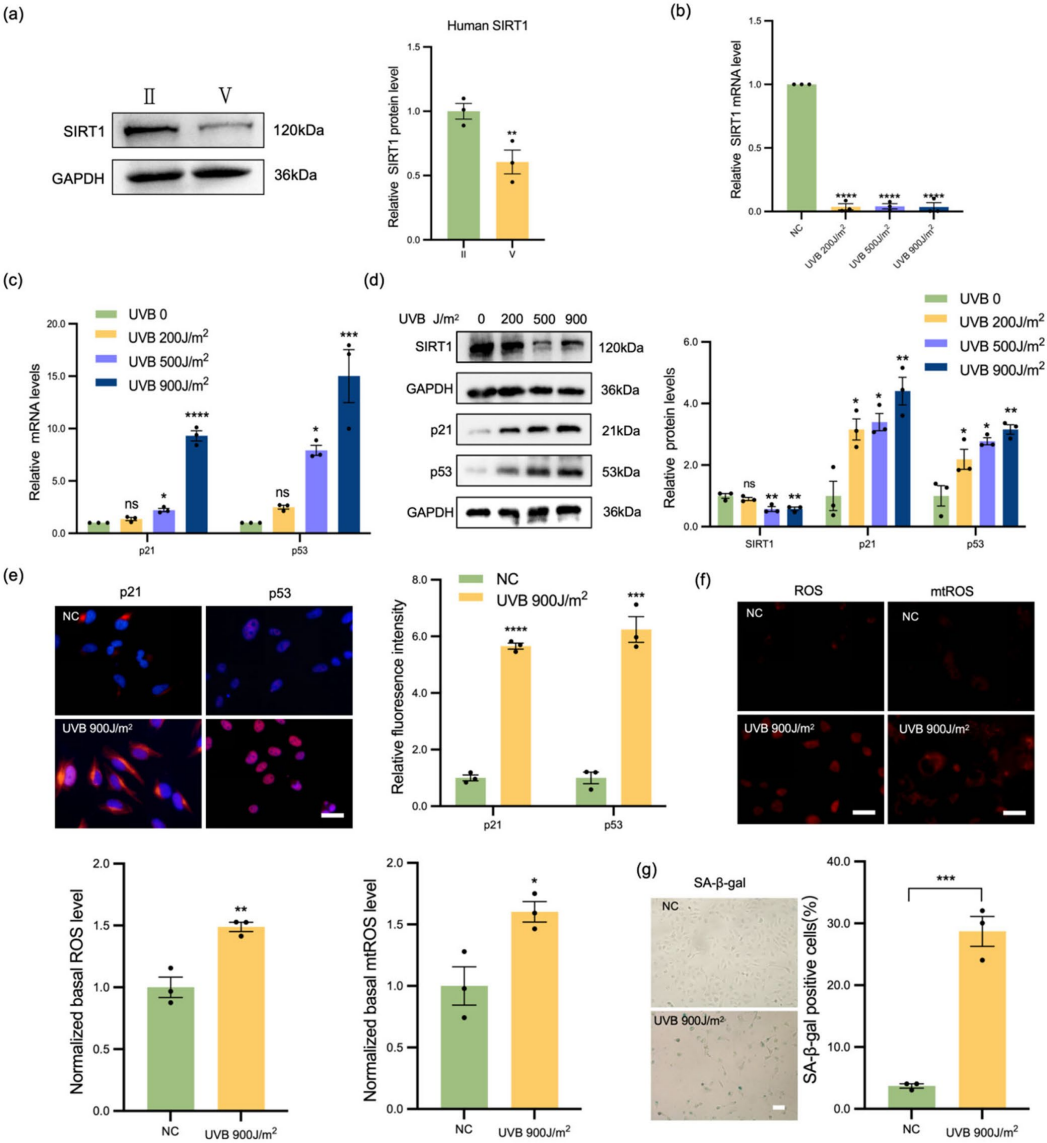
SIRT1 expression was downregulated in cataract. (a) WB analysis of SIRT1 expression in samples from ARC patients diagnosed with grade II (*n* = 10 samples per group) and V (*n* = 10 samples per group). (b,c) qPCR analysis of SIRT1, p21, and p53 expression in LEC after 24 h treated with different UVB doses. (d) WB analysis of SIRT1, p21, and p53 protein expression in LEC after 48 h treated with different UVB doses. (e) IF staining of p21 (red) and p53 (red) merged with DAPI (blue) in each group, 48 h after UVB irradiation. Scale bars = 50 μm. (f) After staining with DHE and MitoSOX Red Dye for 24 h, ROS and mtROS were detected. Scale bars = 50 μm. (g) The percentage of senescent cells 48 h after UVB irradiation was determined by SA‐β‐gal staining. Scale bars = 100 μm. *p < 0.05; **p < 0.01; ***p < 0.001; ****p < 0.0001. Data are shown as means ± SEMs (*n* = 3).

To explore the role of SIRT1 in cataract, we collected the anterior capsule of ARC patients divided them into two groups according to the Emery grading standard, grade II (*n* = 10 samples per group) and grade V (*n* = 10 samples per group), and we analyzed the expression of SIRT1 in the lens samples from the two groups of cataract patients with different degrees of opacity. As shown (Table 1) in Figure 1a, the expression of SIRT1 in the grade V samples was lower than that in the grade II. SA‐β‐gal staining was performed on the lens capsule to detect senescent cells. Representative images of a 49‐year‐old and an 81‐year‐old capsule were shown; cells in the 81‐year‐old capsule exhibited a senescent phenotype, as indicated by positive SA‐β‐gal staining (Figure S1A). In contrast, the number of SA‐β‐gal positive cells in the anterior capsule of the 49‐year‐old patient lens capsular was lower. Further, we observed that the number of SA‐β‐gal positive cells was directly correlated with the age of the lens capsule (Figure S1B). WB and qPCR analyses revealed that UVB irradiation significantly downregulated SIRT1 expression in LEC (Figure 1b,d). Then, LECs were exposed to UVB, after which the expression of aging‐related proteins (p21 and p53) was assessed. We used different intensities of UVB (200, 500, and 900 J/m^2^) to irradiate LEC to induce aging. The expression levels of p21 and p53 in LEC exposed to UVB increased in a dose‐dependent manner (Figure 1c,d). IF results were highly consistent with the WB results, indicating that the expression levels of p21 and p53 significantly increased under UVB stimulation (Figure 1e). In addition, UVB stimulation led to ROS and mtROS accumulation in LEC, as manifested by the increased fluorescence intensity of MitoSOX and DHE (Figure 1f). SA‐β‐gal staining revealed that UVB irradiation promoted the expression of senescence markers (Figure 1g). In summary, we found that SIRT1 was decreased during LEC senescence.

### 3.2 SIRT1 Protected UVB‐Induced LEC Senescence

**FIGURE 2:**
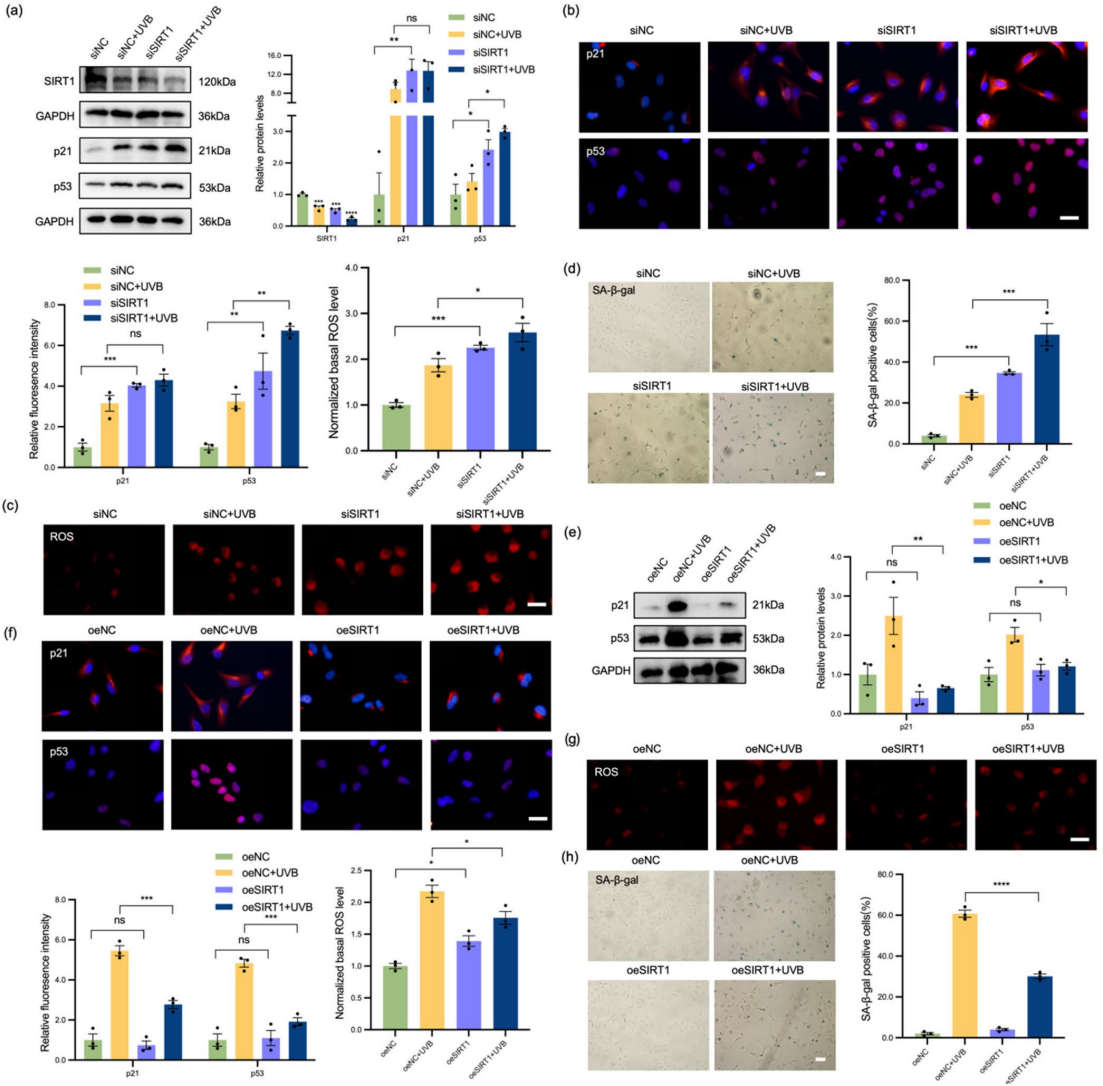
SIRT1 delayed UVB‐induced LEC senescence. (a) WB analysis showed the protein levels of SIRT1, p21, and p53 in the siNC, siNC+UVB, siSIRT1, and siSIRT1 + UVB groups. (b) IF staining of p21 (red) and p53 (red) merged with DAPI (blue) in each group. Scale bars = 50 μm. (c) DHE staining was used to detect the intracellular ROS levels in each group. Scale bars = 50 μm. (d) SA‐β‐gal staining to determine the percentage of senescent cells. Scale bars = 100 μm. (e) WB analysis showed that the protein levels of SIRT1, p21, and p53 in the oeNC, oeNC+UVB, oeSIRT1, and oeSIRT1 + UVB groups. (f) IF staining of p21 (red) and p53 (red) merged with DAPI (blue) in each group. Scale bars = 50 μm. (g) DHE staining was used to detect the intracellular ROS levels in each group. Scale bars = 50 μm. (h) SA‐β‐gal assay was used to analyze the percentage of senescent cells. Scale bars = 100 μm. *p < 0.05; **p < 0.01; ***p < 0.001; ****p < 0.0001. Data are shown as means ± SEMs (*n* = 3).

To determine the role of SIRT1 in LEC senescence, we performed loss‐of‐function studies by blocking SIRT1 expression via siRNA. As shown in Figure S2A,B, siRNA#3 exhibited the highest knockdown efficiency, reducing SIRT1 mRNA by more than 90% and protein levels by approximately 60%; thus, it was selected for subsequent experiments. We then explored the effect of SIRT1 inhibition on LEC under UVB irradiation, and compared with UVB‐treated siNC‐transfected cells, the UVB‐treated SIRT1‐silenced cells showed a more significant increase in p53 accumulation (Figure 2a). IF results confirmed that p21 and p53 expression in LEC increased after SIRT1 inhibition (Figure 2b). Regarding ROS, both siSIRT1 transfection and UVB irradiation significantly increased the intracellular ROS level (Figure 2c). Moreover, the SA‐β‐gal assay revealed that both siSIRT1 transfection and UVB treatment exacerbated the premature senescence of LEC (Figure 2d). We then explored whether upregulation of SIRT1 expression could prevent UVB‐induced LEC senescence. We overexpressed SIRT1 by transfecting cells with SIRT1 plasmids; the overexpression efficiency is shown in Figure S2C,D. As expected, SIRT1 overexpression led to a decrease in p21 and p53 expression in UVB‐induced LEC (Figure 2e). IF results further verified that SIRT1 overexpression slowed LEC senescence (Figure 2f). SIRT1 overexpression slowed down excessive ROS generation (Figure 2g). In addition, the SA‐β‐gal assay revealed that SIRT1 overexpression reduced the proportion of SA‐β‐gal‐positive cells (Figure 2h). These results suggest that SIRT1 promotes LEC survival by protecting LEC from UVB‐induced senescence.

### 3.3 p66Shc Mediated the Effects of SIRT1 on UVB‐Induced Cell Senescence

**FIGURE 3:**
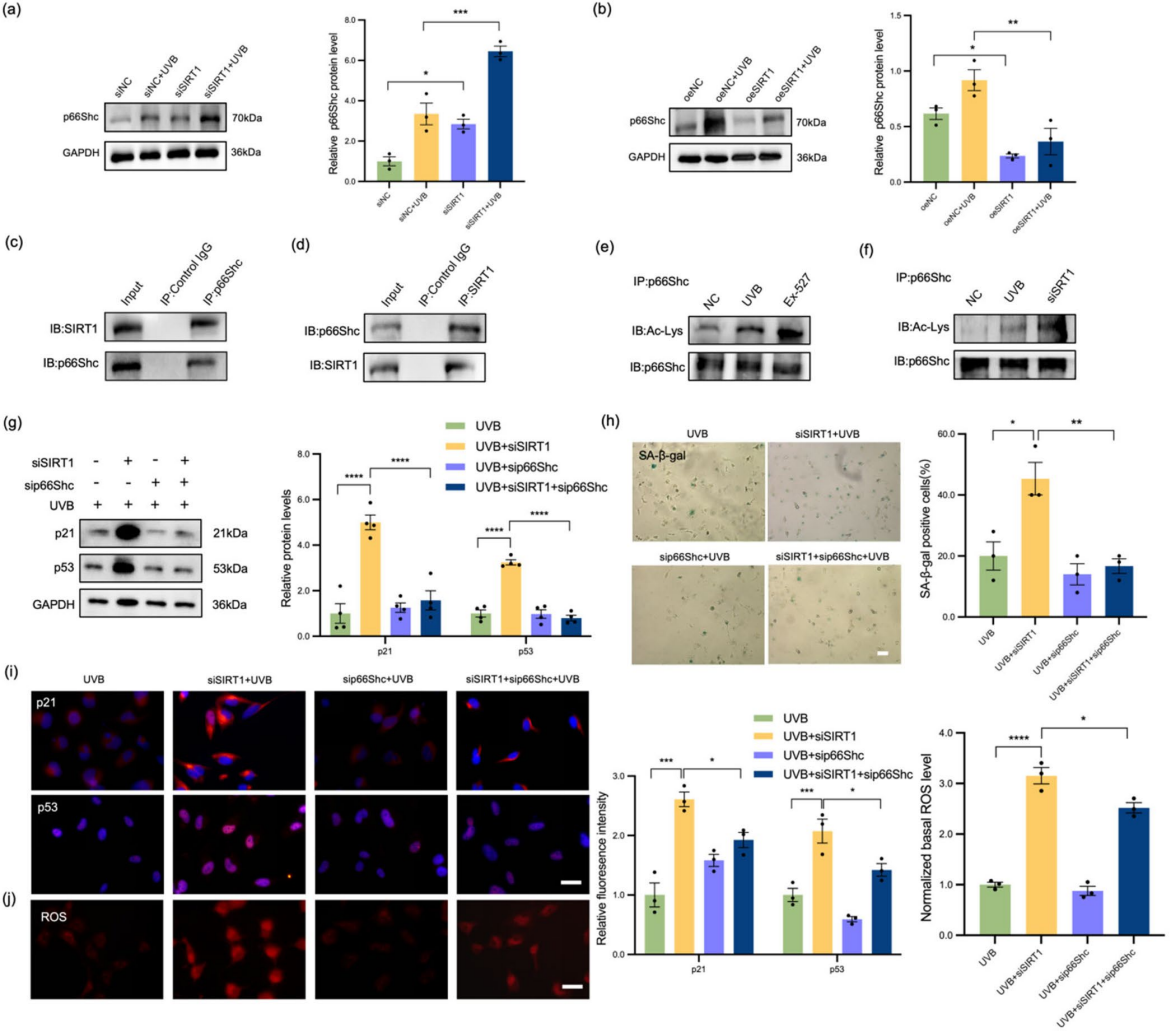
SIRT1 inhibited UVB‐induced LEC senescence by regulating p66Shc deacetylation. (a) WB analysis showed the protein level of p66Shc in the siNC, siNC+UVB, siSIRT1, and siSIRT1 + UVB groups. (b) WB analysis showed the protein level of p66Shc in the oeNC, oeNC+UVB, oeSIRT1, and oeSIRT1 + UVB groups. (c) Co‐IP assay was performed with an anti‐p66Shc antibody, followed by WB analysis of SIRT1 and p66Shc. (d) Co‐IP assay using an anti‐SIRT1 antibody followed by WB analysis of SIRT1 and p66Shc. (e) The acetylation level of p66Shc was assessed by WB. (f) The acetylation of p66Shc detected in HLEB3 cells expressed NC, UVB, and siSIRT1 groups. (g) Effects of the double knockdown of SIRT1 and p66Shc on cell senescence after UVB treatment. The expression of p21 and p53 was assessed by WB. (h,i) IF staining and DHE dye of p21 (red), p53 (red) and nuclei (blue) in the UVB, UVB + siSIRT1, UVB + sip66Shc and UVB + siSIRT1 + sip66Shc groups. Scale bars = 50 μm. (j) The percentage of senescent cells was analyzed by SA‐β‐gal staining. Scale bars = 100 μm. *p < 0.05; **p < 0.01; ***p < 0.001; ****p < 0.0001. Data are shown as means ± SEMs (*n* = 3).

p66Shc is a key transcription factor that regulates oxidative stress and cell senescence (Migliaccio et al. 1999). We performed immunoblot experiments on the collected anterior capsule samples and found that the protein expression level of p66Shc increased with the aggravation of lens opacity (Figure S3A). Subsequent experiments in cell models revealed that UVB irradiation significantly upregulated the mRNA and protein expression levels of p66Shc in a dose‐dependent manner (Figure S3B,C) and that SIRT1 inhibition increased the protein expression of p66Shc (Figure 3a). In contrast, SIRT1 overexpression reduced the protein level of p66Shc (Figure 3b). These results suggest that p66Shc may play an important role in the aging process of LEC. Through protein–protein molecular docking analysis, we found that the binding energy between SIRT1 and p66Shc was −18.1 kcal/mol, indicating that there is a strong binding ability between the two proteins (Figure S4). The physical interaction between SIRT1 and p66Shc was subsequently verified by Co‐IP experiments (Figure 3c,d). To confirm the deacetylation effect of SIRT1 on p66Shc, we assessed the acetylation level of p66Shc under UVB irradiation or the addition of SIRT1 inhibitor Ex‐527 to evaluate the acetylation activity of SIRT1. The results showed that Ex‐527 significantly increased the acetylation level of p66Shc; in addition, UVB irradiation significantly increased the degree of p66Shc acetylation (Figure 3e). Consistently, the knockdown of SIRT1 augmented the acetylation levels of p66Shc (Figure 3f). Next, we explored whether p66Shc is involved in the anti‐aging effect mediated by SIRT1. Silencing p66Shc rescued LEC senescence induced by both SIRT1 knockdown and UVB treatment (Figure 3g–j). We successfully achieved p66Shc overexpression by transfecting p66Shc plasmid, and the overexpression efficiency was shown in Figure S5A,B. In addition, p66Shc overexpression significantly reversed the SIRT1‐mediated inhibition of cell senescence (Figure S6A). IF, ROS detection, and SA‐β‐gal assay results further supported the above findings, indicating that silencing p66Shc alleviated cell senescence caused by SIRT1 deficiency, while p66Shc overexpression exacerbated the senescent phenotype under UVB irradiation (Figure S6B,C). In summary, p66Shc was confirmed to be a key downstream molecule in the SIRT1‐mediated antiaging process. SIRT1 effectively protects LEC from aging damage by regulating the expression of p66Shc.

### 3.4 NMN Treatment Inhibited LEC Senescence via Changes in Mitochondrial Morphology and Function

**FIGURE 4:**
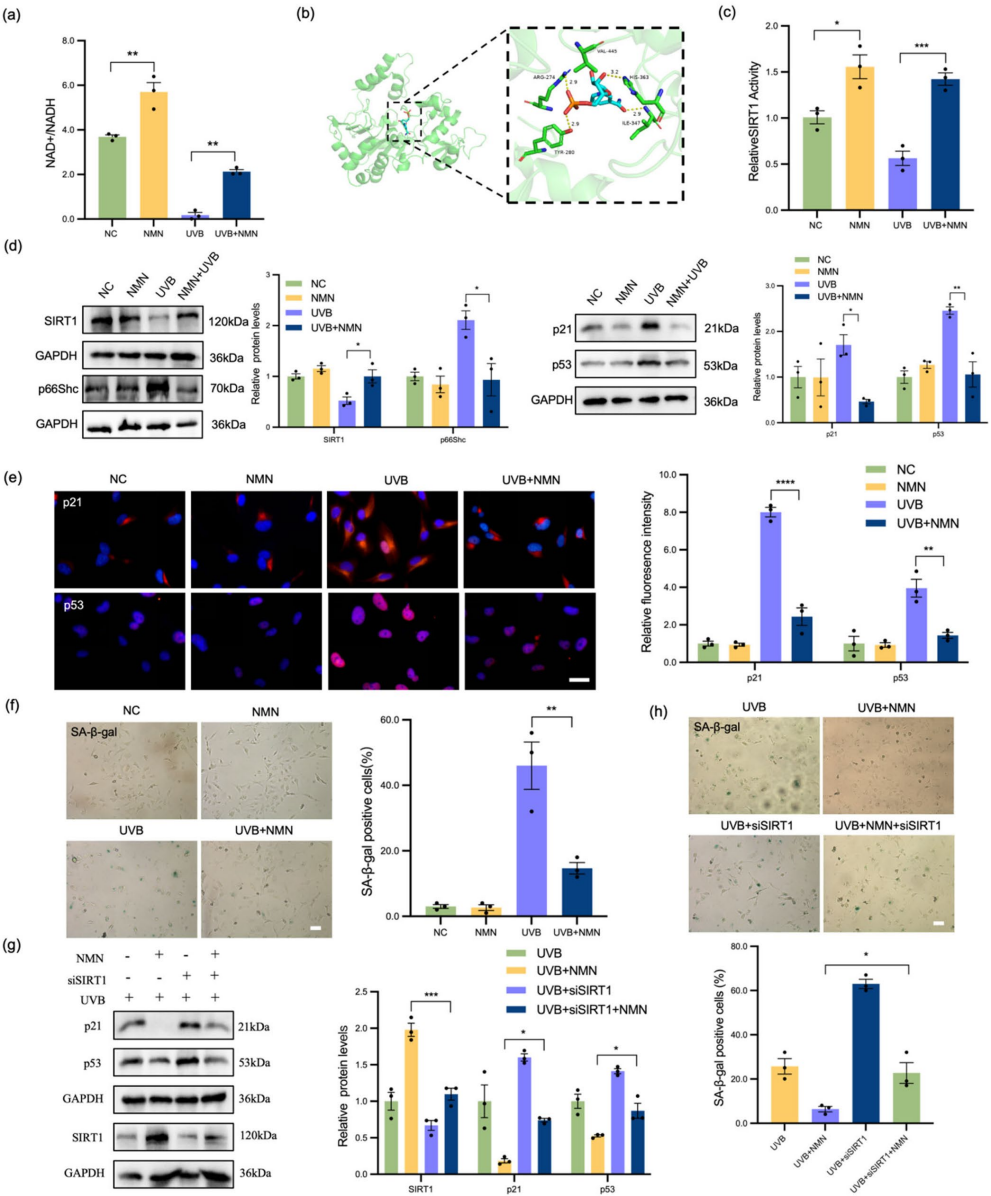
NMN treatment delayed UVB‐induced LEC senescence. (a) NMN increased the intracellular NAD+ level in the UVB‐irradiated cell model. (b) Schematic diagram of the three‐dimensional structure of the interaction between NMN and SIRT1 and the enlarged details. (c) The enzymatic activity of SIRT1 was detected in LEC. (d) WB analysis showed that the protein levels of SIRT1, p21, and p53 in the NC, NMN, UVB, and UVB + NMN groups. (e) IF staining of p21 (red) and p53 (red) merged with DAPI (blue) in each group. Scale bars = 50 μm. (f) SA‐β‐gal staining to determine the percentage of senescent cells. Scale bars = 100 μm. (g) Relative protein expression levels of aging markers in different groups were assessed by WB. (h) The percentage of senescent cells was analyzed by SA‐β‐gal staining. Scale bars = 100 μm. *p < 0.05; **p < 0.01; ***p < 0.001. Data are shown as means ± SEMs (*n* = 3).

NMN is considered one of the most representative NAD+ precursors. In the cellular environment, NMN can be converted into NAD+. Therefore, we explored whether NMN provides protection to cells by increasing the level of cellular NAD+. The results of NAD+/NADH content analysis revealed that NMN treatment significantly increased the level of intracellular NAD+ regardless of UVB treatment (Figure 4a), indicating that NMN may protect LEC from damage by promoting the generation of NAD+. Molecular docking simulations revealed that NMN binds to SIRT1 through multiple hydrogen bonds and hydrophobic interactions, enhancing the stability of NMN in the SIRT1 protein binding pocket to form a stable complex (Figure 4b). Since the anti‐senescence effect of SIRT1 depends on its enzymatic activity and may be regulated by NAD+ enhancers, we used fluorescent signal tracking to detect the deacetylation activity of peptide substrates. NMN significantly increased the enzymatic activity of SIRT1 in cells (Figure 4c). In addition, NMN reduced the expression levels of UVB‐induced senescence‐related proteins (p21 and p53) (Figure 4d), and IF results were consistent with the WB results (Figure 4e). Under UVB treatment, NMN effectively alleviated the senescence of LEC and reduced the proportion of SA‐β‐gal positive cells (Figure 4f). Notably, when SIRT1 was silenced, the anti‐aging effect of the NAD+ supplement NMN was significantly weakened (Figure 4g,h), further supporting the core role of SIRT1 in NMN's anti‐senescence mechanism.

**FIGURE 5:**
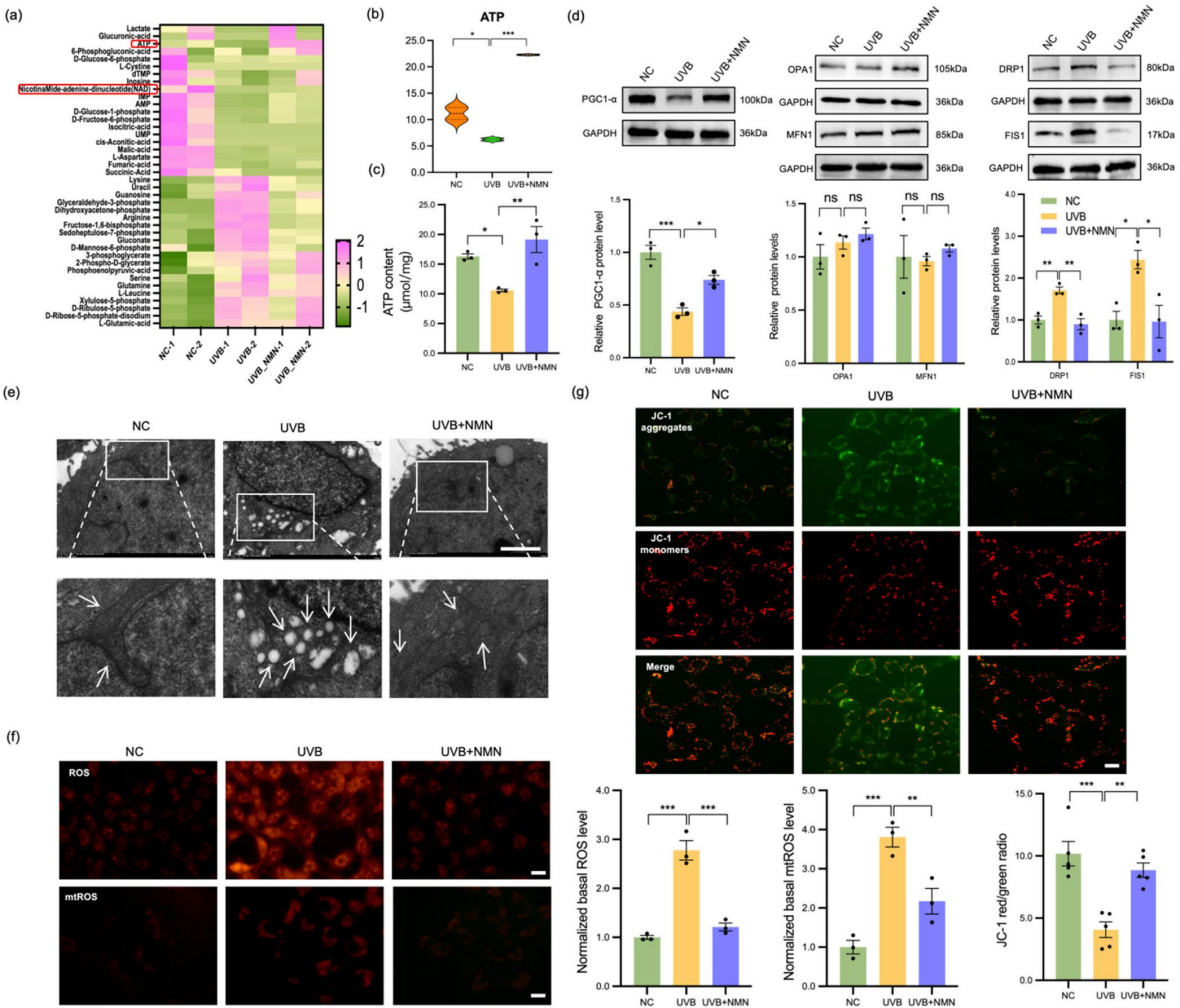
NMN prevented mitochondrial dysfunction in UVB‐treated LEC. (a) Representative heatmap from hierarchical clustering analysis showing significant differences in metabolites among the groups (*n* = 2). (b) Violin plot of ATP changes in the NC, UVB, and UVB + NMN groups. (c) ATP abundance levels in the NC, UVB, and UVB + NMN groups. (d) Protein levels of the mitochondrial dynamic genes PGC1‐α, DRP1, FIS1, OPA1, and MFN1 were assessed by WB. (e) LEC mitochondria were imaged by TEM. Scale bar = 2 μm. (f) ROS and mtROS were detected by DHE and MitoSOX dye. Scale bar = 20 μm. (g) MMP was measured by JC‐1 staining. (*n* = 5 fields per groups) Scale bar = 20 μm. *p < 0.05; **p < 0.01; ***p < 0.001. Data are shown as means ± SEMs (*n* = 3).

We then explored the effects of UVB irradiation and NMN intervention on the abundance of metabolites in LEC. Heatmap analysis (Figure 5a) revealed that there were significantly different metabolites among the control group, UVB group, and UVB + NMN group. Among alterations in metabolites, the changes in ATP and NAD were particularly significant. NMN treatment significantly altered the abundance pattern of ATP (Figure 5b,c), suggesting that NMN plays an important role in the regulation of energy metabolism.

Mitochondrial dysfunction plays an important pathogenic role in the occurrence and development of cataract. To evaluate whether UVB can induce mitochondrial dysfunction and the protective effect of NMN against this process. To further study the changes in mitochondrial dynamics, we analyzed marker proteins related to mitochondrial generation and fission and fusion by WB. The UVB‐treated group presented a significant decrease in mitochondrial generation capacity and enhanced mitochondrial fission but no significant change in mitochondrial fusion (Figure 5d). We used TEM to visualize mitochondria and found that the mitochondria in the control group were rod‐shaped and elongated, whereas those in the UVB‐treated group were swollen, fragmented, and lacked ridges. Notably, in the NMN‐treated group, UVB‐induced reduction in mitochondrial swelling was observed (Figure 5e). By measuring total cellular ROS and mtROS levels, we found that UVB caused excessive ROS and mtROS accumulation in LEC, an effect manifested by a significant increase in the fluorescence intensity of DHE and MitoSOX (Figure 5f). In addition, we used JC‐1 staining to evaluate the effect of UVB on MMP, and the results revealed that the MMP significantly decreased after UVB treatment (Figure 5g). Notably, NMN treatment not only significantly increased the MMP level and reduced the excessive accumulation of mtROS, but also restored the mitochondrial generation capacity and inhibited the excessive fission of mitochondria. In addition, NMN reversed the reduced mitochondrial generation and increased fission caused by SIRT1 knockdown (Figure S7A–C). These results indicate that UVB‐induced MMP abnormalities and oxidative stress responses are important mechanisms of LEC damage. NMN effectively protects mitochondrial function and alleviates UVB‐induced damage by increasing the expression level of SIRT1. These findings provide evidence of the potential application value of NMN as a mitochondrial protector in the pathological process of UVB‐induced cataract.

### 3.5 NMN Attenuated Lens Transparency in the UVB‐Treated Rat

**FIGURE 6:**
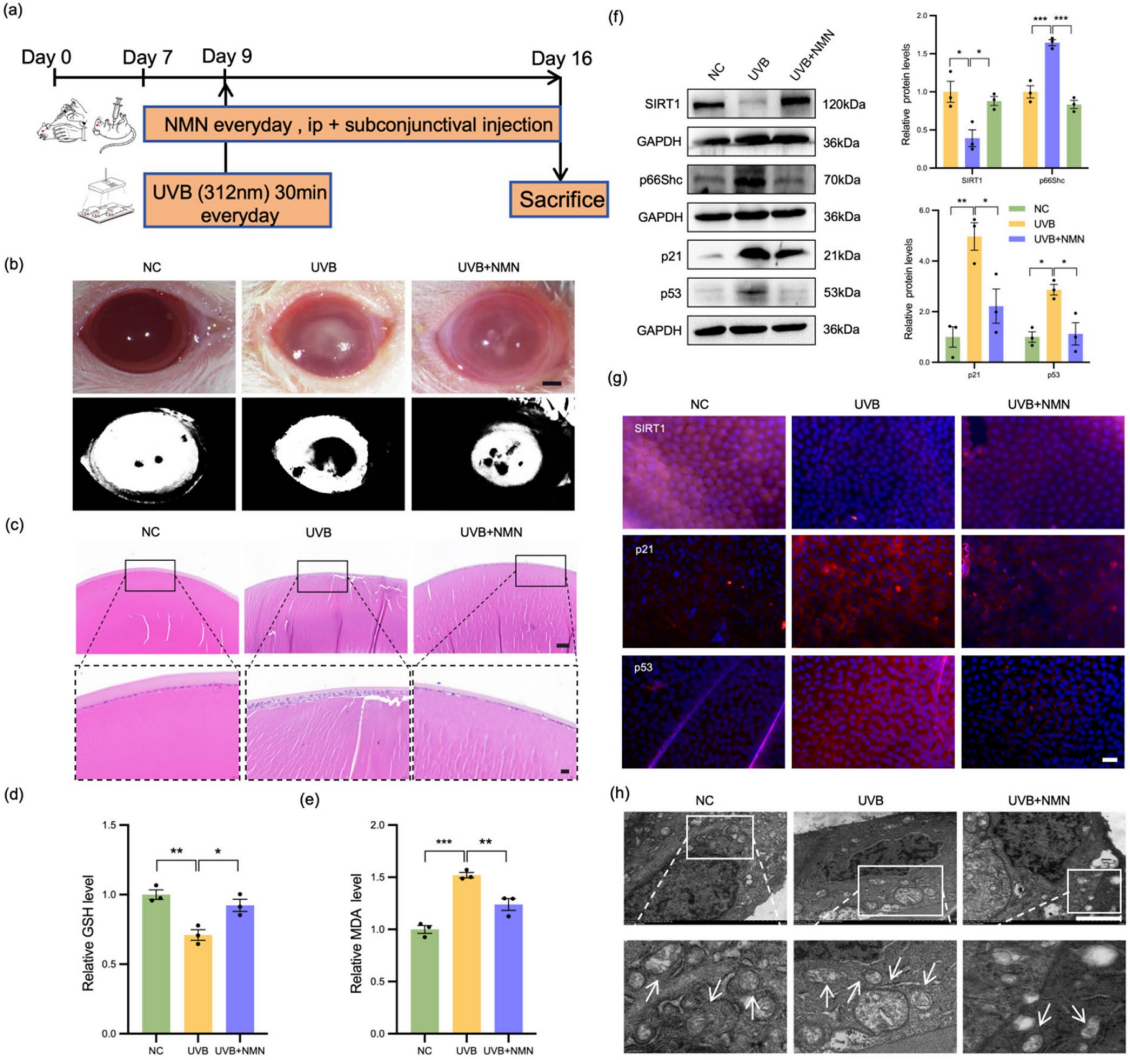
NMN alleviated UVB‐induced lens transparency in rats. (a) Schematic representation of the SD rat model. (b) Observation of rat lenses in different groups under a stereomicroscope and their color threshold segmentation images. Scale bar = 1 mm. (c) Total lens, HE staining. Scale bars = 100 μm. (d,e) Analysis of the expression levels of markers of oxidative stress in the lenses of rats. (f) WB analysis showed the protein levels of SIRT1, p66Shc, p21 and p53 in the lenses of the rats in each group. (g) IF staining of SIRT1 (red), p21 (red) and p53 (red) merged with DAPI (blue) in rat lenses. Scale bars = 20 μm. (h) Mitochondria in the anterior capsule region of the lenses of rats were imaged by TEM. Scale bars = 2 μm. *p < 0.05; **p < 0.01; ***p < 0.001. Data are shown as means ± SEMs (*n* = 3).

Based on the results of in vitro experiments, we confirmed the significant role of NMN in preventing cell senescence and protecting mitochondrial dysfunction in UVB‐induced cell models. To further investigate the protective effect of NMN, we established the rat ARC model by irradiating the rat lens with UVB light to simulate the occurrence of cataract (Figure 6a). The area of clouding in the rat lens observed by light microscopy after UVB exposure and drug pretreatment is shown in Figure 6A. Lenses were extracted from SD rats, and those in the UVB group presented obvious opacities (Figure S8). The histological staining results revealed that the LEC in the control group were arranged regularly with consistent cell spacing, whereas the LEC in the UVB‐treated group presented a disordered arrangement, irregular cell spacing, vacuoles, swelling and disordered proliferation, in addition to significant changes in nuclear morphology (Figure 6c). Oxidative stress has been shown to be closely related to cell senescence and is an important cause of lens opacity. Therefore, we assessed the levels of markers of oxidative stress (such as GSH and MDA) in rat lenses. UVB irradiation significantly altered the levels of antioxidant oxidase in the lens (Figure 6d,e). IF staining and WB analysis of the anterior lens capsule revealed that senescence markers (p21 and p53) accumulated in the anterior lens capsule, whereas the expression level of SIRT1 was significantly decreased (Figure 6f,g). Encouragingly, NMN treatment attenuated UVB‐induced LEC senescence, significantly reduced the formation of lens white spots (Figure 6b) and increased the levels of markers of oxidative stress (Figure 6d,e), thereby maintaining the monolayer cubic arrangement structure of LEC and protecting the transparency of the lens. Notably, the addition of NMN largely reversed cataract formation in UVB‐exposed lenses (Figure S8). These results indicate that NMN has a significant protective effect against UVB radiation by increasing the antioxidant capacity and slowing the aging process. Next, we evaluated the role of mitochondria in the development of ARC and the protective effect of NMN. The ultrastructure of mitochondria in the anterior lens capsule region after UVB irradiation was observed by TEM, and in UVB‐treated group, the mitochondria swelling and damage were aggravated and the cell nucleus was condensated. In contrast, NMN treatment significantly improved the ultrastructure of mitochondria and alleviated UVB‐induced damage (Figure 6h). In summary, these findings suggest that NMN can effectively alleviate UVB‐induced lens damage and slow cataract development by increasing antioxidant capacity and protecting mitochondrial function.

## 4 Discussion

In this study, NMN treatment delayed oxidative stress‐induced LEC senescence both in vivo and in vitro. We evaluated the regulatory effects of SIRT1 and p66Shc on the senescence phenotype of LEC through loss‐of‐function and gain‐of‐function experiments. Using metabolite sequencing, we highlighted energy metabolites, such as ATP, as pivotal in LEC senescence. Further exploration of anti‐aging therapies for lens preservation in ARC is necessary (Figure S9).

In the anterior capsule of the human lens, we observed that the number of senescent cells increased with age, indicating that age‐related changes in the capsule contribute to LEC senescence. Our observation is analogous to several previous studies that have demonstrated an age‐dependent increase in senescent cells (Cooksley et al. 2024). Our study demonstrated that UVB induces LEC senescence, as indicated by a significant upregulation of p21 and p53. Our observation is analogous to several previous studies that have demonstrated UVB‐induced senescence of other cell types (Liu, Ren, et al. 2025; Liu, Xu, et al. 2025; Chou et al. 2013). For example, in human dermal fibroblasts, UVB irradiation caused cellular senescence, which was 5ʹ‐tiRNA‐His‐GTG‐dependent (Liu, Ren, et al. 2025), and in the retina, UVB induced cell cycle arrest by activating the PI3K/AKT/ERK pathway (Chou et al. 2013).

Given that SIRT1 gradually decreases in human lens capsules with aging (Lin et al. 2011), this led us to investigate whether SIRT1 causes LEC senescence. We first investigated the changes of SIRT1 in patients with different stages of cataract. We divided the patients with cataract into mild and severe groups and found that the expression level of SIRT1 in lens samples of patients with severe cataract was significantly reduced. However, there are differences between this result and the study by Zhang et al. Zhang's research has shown that SIRT1 expression is increased in the anterior capsule membranes of the lenses in patients with ARC compared with older but normal lenses (Zheng and Lu 2011). We believe that this difference is mainly due to the different sample classification methods and the influence of oxidative stress response at different stages of cataract on the effect of SIRT1. Additional experiments are needed to test our hypothesis further. Next, the regulatory effects of SIRT1 on UVB‐induced ARC phenotype were further evaluated through loss‐of‐function or gain‐of‐function experiments. We also found that even in the absence of UVB exposure, SIRT1 knockdown can directly induce the generation of ROS and promote LEC senescence, whereas SIRT1 overexpression can effectively prevent the transition of LECs to senescence, indicating that endogenous SIRT1 plays an essential role in maintaining LEC stability and transparency by inhibiting aging.

p66Shc, a mitochondrial adaptor protein, plays a crucial role in the regulation of ROS production and mitochondrial function, requiring its phosphorylation for activation (Pinton et al. 2007). This process produces hydrogen peroxide as a byproduct, which is a major source of ROS (Giorgio et al. 2005). p66Shc is also a stress‐responsive protein, and its functional activity is closely related to its mitochondrial localization (Lebiedzinska‐Arciszewska et al. 2024). Oxidative stimulus‐induced lysine acetylation of p66Shc facilitates its phosphorylation on serine 36 and translocation to the mitochondria, where it promotes hydrogen peroxide production (Kumar et al. 2017). These post‐translational modifications are believed to play an important role in modulating the function of p66Shc in response to cellular stress. Our findings demonstrate that SIRT1 inhibits the pro‐oxidative stress function of p66Shc by deacetylation, thereby maintaining mitochondrial homeostasis and slowing down LEC aging phenotypes. This suggests that the acetylation of p66Shc may be involved in the regulatory process of its aging‐related functions. Although we have not yet constructed acetylation mimetic or inhibitory mutants to directly verify the role of this modification in regulating the function, the results of this study provide new clues for further exploration of the role of acetylation in the signal transduction and cell senescence of p66Shc. In future studies, site‐directed mutagenesis strategies can be adopted to further clarify its mechanism of action.

Our study showed that NMN activates SIRT1 activity by increasing intracellular NAD+ levels, thereby protecting LECs from UVB‐induced cell senescence and maintaining lens transparency in rats. This discovery that NMN is involved in various anti‐aging processes has prompted extensive investigations aimed at delaying cellular aging and preventing age‐related diseases (Rahman et al. 2024; Nadeeshani et al. 2021; Kiss et al. 2019). Based on these results, we have proposed a mechanism by which NMN can be used as a therapeutic tool to support the lens and prevent cataract. NMN, as a NAD+ precursor anti‐aging supplement, has shown considerable promise (Sun et al. 2024; Sauve et al. 2023). In practical applications, how to effectively deliver NMN to the eyes, especially to the lens area, is a key challenge for its transformation into a therapeutic method. Currently, various ocular delivery strategies have been explored, including eye drops, nanoparticle delivery systems, ocular gels, and more penetrating intraocular injection techniques (Loiseau et al. 2025; Dong et al. 2025; Fialho 2025; Yabhouni et al. 2024). Due to the fact that the lens is surrounded by a capsule and has scarce blood vessels, systemic administration (such as oral administration) may have problems with low bioavailability (Chen et al. 2024). Therefore, local delivery methods may be more feasible and efficient. Future research can focus on exploring NMN sustained‐release delivery systems based on nanocarrier‐based or polymer nanoparticles (such as PLGA), in order to enhance their penetration, stability, and duration of action in ocular tissues (Fialho 2025; Yabhouni et al. 2024; Slavkova et al. 2025). At the same time, measures such as combining penetration enhancers and modifying carrier materials can be considered to improve the bioavailability and lens‐targeting ability of NMN (Santos et al. 2025). Moreover, with the development of biodegradable materials and intelligent controlled‐release systems, the possibility of precise delivery and long‐term prevention and treatment of cataracts with NMN will continue to increase (Liu, Ren, et al. 2025).

In our study, we also found that NMN mitigates UVB‐induced declines in MMP, prevents excessive mtROS production, repairs damaged mitochondrial structures, and restores ATP synthesis. It also preserves cellular metabolic function by improving mitochondrial homeostasis through reducing excessive fission and promoting mitochondrial biogenesis. To further test our hypothesis that NMN exerts anti‐aging effects by improving mitochondrial function and cellular energy status, we employed metabolomics to detect the changes in metabolite levels in each group. Energy metabolism analysis showed that the changes in ATP levels were the most significant in the NC, UVB, and NMN treatment groups, suggesting that it might play a crucial role in the UVB‐induced aging and the NMN intervention process. As the most fundamental energy molecule in cells, ATP directly reflects mitochondrial functional status. When mitochondrial damage occurs, the decrease in MMP, structural damage, and accumulation of mtROS will significantly inhibit the oxidative phosphorylation process, resulting in a reduction in ATP synthesis efficiency and thereby inducing cellular dysfunction (Bhargava and Schnellmann 2017). NMN can significantly increase the intracellular level of NAD+, thereby activating NAD+ ‐dependent enzymes such as SIRT1, and subsequently regulating mitochondrial biogenesis, antioxidant response, and energy metabolism (Li et al. 2023; Mouchiroud et al. 2013). Therefore, we speculate that NMN exerts its anti‐aging effect through the SIRT1‐mitochondrial axis, and the restoration of ATP may be a direct manifestation of its downstream effects.

Collectively, our results demonstrate that UVB exposure induces a reduction in SIRT1, activating the upregulation of p66Shc and leading to increased mitochondrial dysfunction, inducing cellular oxidative stress and premature aging. While this study has yielded significant findings, there are some limitations, such as no validation of the apoptosis or necrosis in LECs. Understanding the impact of the SIRT1/p66Shc pathway on mitochondrial dysfunction is imperative. We will further explore the mechanism through the construction of mutants in our subsequent research. Furthermore, the lack of optimization in the UVB exposure model and the potential damage to the cornea require further investigation.

## 5 Conclusion

This study elucidates the key role of the SIRT1/p66Shc pathway in UVB‐induced LEC aging. The results showed that UVB exposure inhibited the deacetylation activity of SIRT1 and increased the acetylation level of p66Shc, resulting in a large accumulation of ROS, thereby damaging mitochondrial structure and function, accelerating the aging of LEC and promoting the occurrence and development of ARC. Furthermore, NMN can effectively improve mitochondrial function by activating SIRT1 and delay the process of LEC aging and lens opacity, providing a new potential strategy for ARC prevention and treatment.

## Author Contributions

Hongyan Ge, Yu Mi, and Huirui Liu designed the study. Huirui Liu, Yi Gao, Fengchun Kang, and Yujing Bai conducted in vitro experiments; Huirui Liu and Xiaohan Yu conducted in vivo experiments. Huirui Liu and Jialin Luo performed statistical analysis on the data. Liyao Sun guided and supervised this study. Huirui Liu wrote the manuscript. Hongyan Ge and Liyao Sun revised the manuscript. All authors have contributed to the submitted version of the manuscript.

## Conflicts of Interest

The authors declare no conflicts of interest.

## Supporting information


Appendix S1.

## Data Availability

All human experiments (Approval Number: No. 2024240) and animal experiments (Approval Number: No. 2023130) are approved by the Ethics Committee of the First Affiliated Hospital of Harbin Medical University. All participants signed written informed consent. The data that support the findings of this study are available from the corresponding author upon reasonable request.
