# Supplementary material for: SIRT1 Prevents Lens Epithelial Cell Senescence During Age‐Related Cataract via Regulating p66Shc
Source: Aging Cell. 2025 Jun 26;24(9):e70155. doi: 10.1111/acel.70155 (PMC12419841; doi:10.1111/acel.70155)
Supplement: Supplementary file 1 — Appendix S1. [file ACEL-24-e70155-s001.docx]

**Table S1. Patient demographics with ARCs**

| Samples | Sex | Age (y) | LOCSⅢ | Emery grading system |
| --- | --- | --- | --- | --- |
| No.1 | Male | 60 | C3N1P1 | Ⅱ |
| No.2 | Male | 57 | C2N0P1 | Ⅱ |
| No.3 | Female | 53 | C3N1P1 | Ⅱ |
| No.4 | Male | 61 | C3N1P0 | Ⅱ |
| No.5 | Female | 50 | C3N0P1 | Ⅱ |
| No.6 | Female | 56 | C2N1P1 | Ⅱ |
| No.7 | Male | 53 | C2N0P0 | Ⅱ |
| No.8 | Female | 57 | C3N1P1 | Ⅱ |
| No.9 | Female | 61 | C3N1P1 | Ⅱ |
| No.10 | Female | 61 | C2N0P1 | Ⅱ |
| No.11 | Male | 78 | C3N1P1 | Ⅱ |
| No.12 | Male | 75 | C2N1P1 | Ⅱ |
| No.13 | Female | 72 | C2N1P1 | Ⅱ |
| No.14 | Male | 79 | C2N1P1 | Ⅱ |
| No.15 | Female | 65 | C3N1P1 | Ⅱ |
| No.16 | Female | 78 | C2N1P1 | Ⅱ |
| No.17 | Female | 74 | C2N1P1 | Ⅱ |
| No.18 | Male | 75 | C2N1P1 | Ⅱ |
| No.19 | Female | 79 | C2N1P1 | Ⅱ |
| No.20 | Female | 68 | C2N1P1 | Ⅱ |
| No.21 | Male | 61 | C2N1P1 | Ⅱ |
| No.22 | Male | 52 | C2N0P1 | Ⅱ |
| No.23 | Female | 58 | C2N1P1 | Ⅱ |
| No.24 | Male | 60 | C3N1P0 | Ⅱ |
| No.25 | Male | 50 | C2N0P1 | Ⅱ |
| No.26 | Female | 56 | C3N1P0 | Ⅱ |
| No.27 | Female | 54 | C2N0P0 | Ⅱ |
| No.28 | Female | 57 | C3N1P1 | Ⅱ |
| No.29 | Female | 62 | C3N1P0 | Ⅱ |
| No.30 | Male | 61 | C2N0P1 | Ⅱ |
| No.31 | Male | 80 | C5N3P1 | Ⅴ |
| No.32 | Male | 57 | C6N3P1 | Ⅴ |
| No.33 | Male | 56 | C6N4P1 | Ⅴ |
| No.34 | Female | 71 | C5N3P0 | Ⅴ |
| No.35 | Male | 60 | C5N3P1 | Ⅴ |
| No.36 | Female | 56 | C5N3P1 | Ⅴ |
| No.37 | Female | 58 | C5N3P0 | Ⅴ |
| No.38 | Male | 53 | C5N4P1 | Ⅴ |
| No.39 | Female | 73 | C6N3P1 | Ⅴ |
| No.40 | Female | 61 | C6N3P0 | Ⅴ |
| No.41 | Male | 68 | C5N3P1 | Ⅴ |
| No.42 | Male | 71 | C5N3P1 | Ⅴ |
| No.43 | Male | 62 | C6N4P1 | Ⅴ |
| No.44 | Male | 59 | C5N2P0 | Ⅴ |
| No.45 | Male | 55 | C5N3P1 | Ⅴ |
| No.46 | Female | 68 | C5N3P1 | Ⅴ |
| No.47 | Female | 64 | C5N3P0 | Ⅴ |
| No.48 | Male | 75 | C6N3P1 | Ⅴ |
| No.49 | Female | 80 | C6N3P1 | Ⅴ |
| No.50 | Female | 58 | C6N3P1 | Ⅴ |
| No.51 | Male | 62 | C6N4P1 | Ⅴ |
| No.52 | Female | 57 | C6N4P1 | Ⅴ |
| No.53 | Male | 54 | C6N4P1 | Ⅴ |
| No.54 | Male | 67 | C5N3P0 | Ⅴ |
| No.55 | Male | 50 | C5N3P1 | Ⅴ |
| No.56 | Female | 59 | C5N3P1 | Ⅴ |
| No.57 | Male | 57 | C6N4P0 | Ⅴ |
| No.58 | Female | 57 | C5N4P1 | Ⅴ |
| No.59 | Female | 64 | C5N5P1 | Ⅴ |
| No.60 | Female | 66 | C6N5P1 | Ⅴ |
| No.61 | Male2 | 49 | C3N1P1 | Ⅱ |
| No.62 | Male25 | 65 | C5N4P1 | Ⅴ |
| No.63 | Male55 | 81 | C5N3P1 | Ⅴ |
| No.64 | Female11 | 56 | C5N4P1 | Ⅴ |
| No.65 | Male50 | 87 | C6N4P1 | Ⅴ |
| No.66 | Female13 | 54 | C2N1P1 | Ⅱ |
| No.67 | Female32 | 68 | C6N5P1 | Ⅴ |

**Table S2. Primer sequences for qPCR**

| **Gene** | **Primer sequences** |
| --- | --- |
| SIRT1 | Forward: ATTGGGTACCGAGATAACCTTCTGT  Reverse: GTATATGGACCTATCCGTGGCCTT |
| p21 | Forward: AGGTGGACCTGGAGACTCTCAG  Reverse: TCCTCTTGGAGAAGATCAGCCG |
| p53 | Forward: CCTCAGCATCTTATCCGAGTGG  Reverse: TGGATGGTGGTACAGTCAGAGC |
| p66Shc | Forward: ACAGCCGAGTATGTCGCCTATG  Reverse: CAATGGTGCTGATGACATCCTGG |
| GAPDH | Forward: CCATGTTCGTCATGGGTGTGAA  Reverse: GGCATGGACTGTGGTCATGAG |

**Table S3. Primary antibodies for WB assay**

| **Antibody name** | **Dilutions** | **Vendor name** | **Cat No.** |
| --- | --- | --- | --- |
| SIRT1 | 1:1000 | CST | 8469s |
| p21 | 1:2000 | Proteintech | 10355-1-AP |
| p53 | 1:10000 | Proteintech | 10442-1-AP |
| p66Shc | 1:1000 | Affinity | AF6245 |
| PGC1-α | 1:1000 | ABclonal | A12348 |
| Drp1 | 1:1500 | Immuno way | YN3054 |
| FIS1 | 1:1000 | Immuno way | YN5292 |
| MFN1  OPA1 | 1:1500  1:1000 | Immuno way  Immuno way | YN3054  YN2976 |
| GAPDH | 1:2000 | OriGene | TA802519 |
| Acetylated-Lysine | 1:1000 | CST | 9441 |

Figure S1. Senescent cells have been identified in the lens anterior capsules of patients. (A) Representative image shows SA-β-gal positive aged cells from the lens anterior capsular samples of individuals aged 49 and 87 years old. (B) Seven lens anterior capsular samples from patients aged 49 to 87 were subjected to SA-β-gal staining. The percentage of senescent cells was determined. (*n*=7) Scale bars = 50μm.


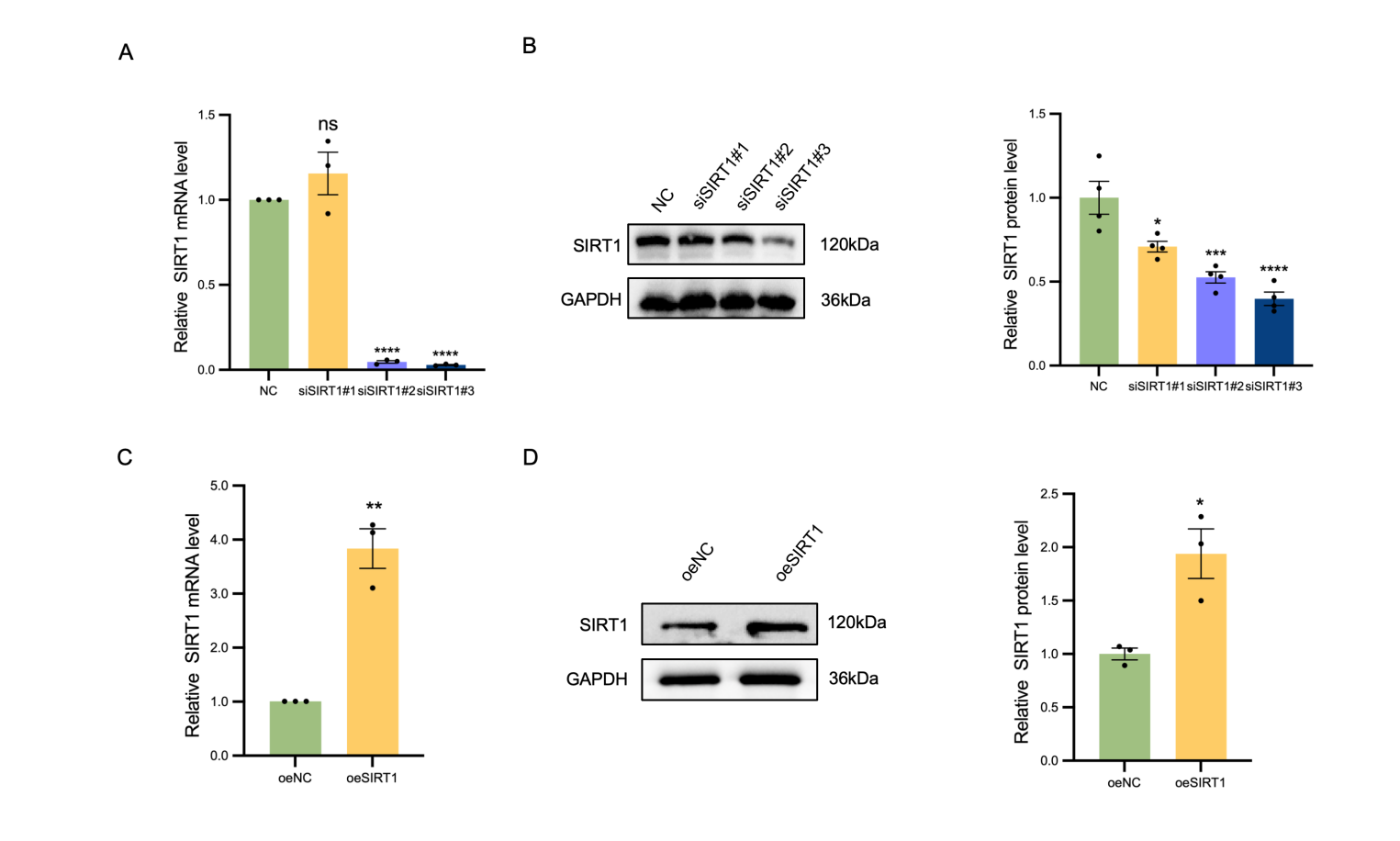


Figure S2. siRNA and Plasmid transfection efficiency of SIRT1. (A) SIRT1 siRNA transfection efficiency was assessed by qPCR. (B) SIRT1 siRNA transfection efficiency was assessed by WB. (C) oeSIRT1 transfection efficiency was assessed by qPCR. (D) oeSIRT1 transfection efficiency was assessed by WB.


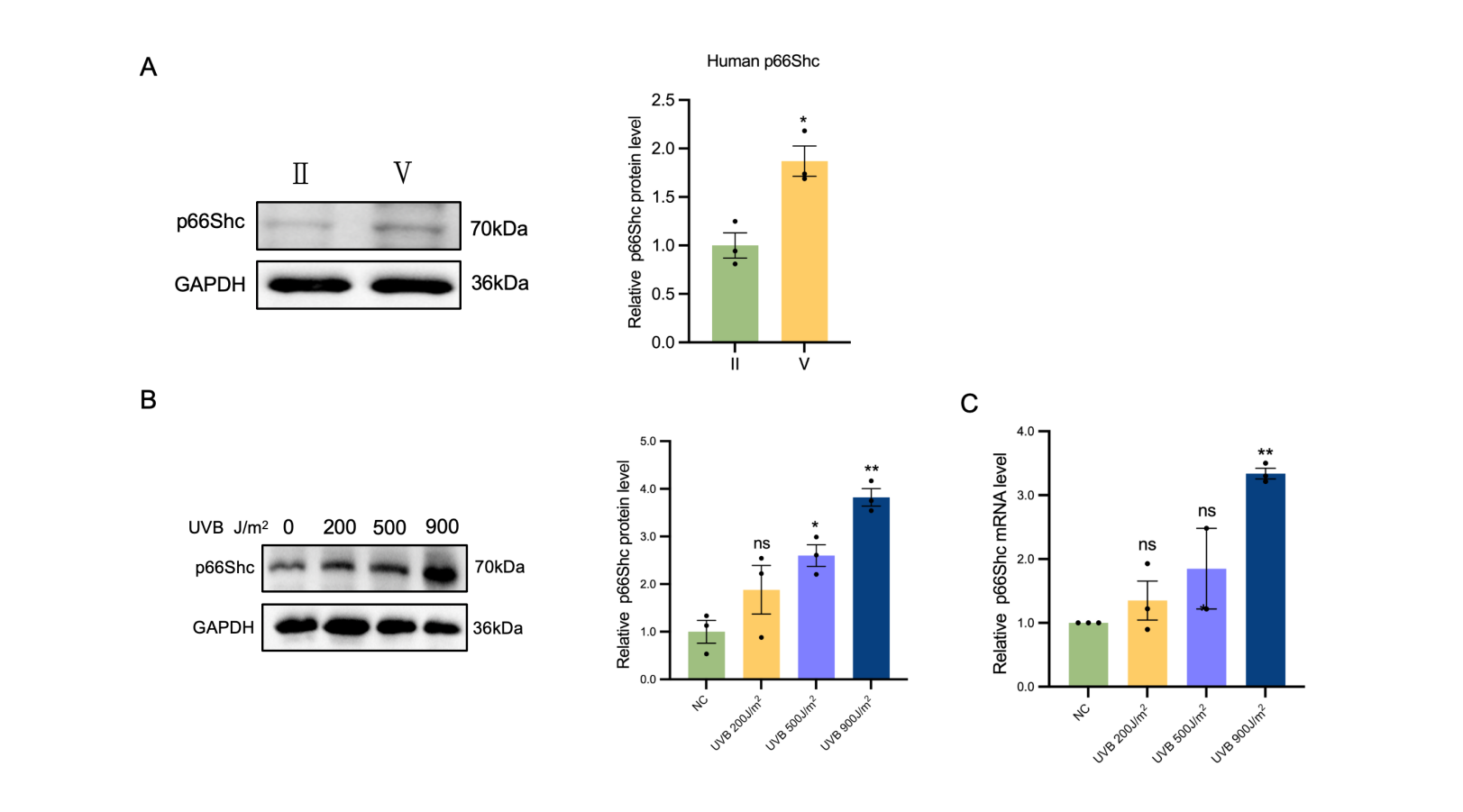


Figure S3. p66Shc was up-regulated in cataract. (A) WB analysis of SIRT1 expression in samples from ARC patients diagnosed with nuclear sclerosis grade II (*n* = 10 samples per group) and V (*n* = 10 samples per group). (B,C) qPCR and WB analyses of mRNA and protein level of p66Shc in LECs treated with UVB (200 J/m^2^, 500 J/m^2^, and 900 J/m^2^).


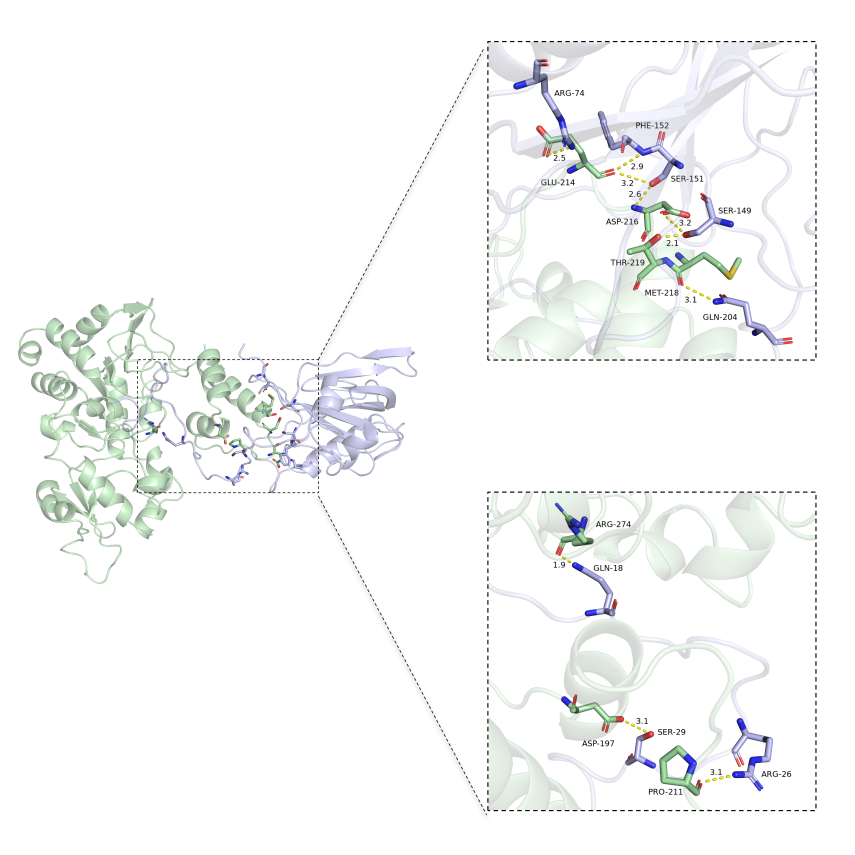


Figure S4. Schematic diagram of the three-dimensional structure of the interaction between SIRT1 and p66Shc and the enlarged details.


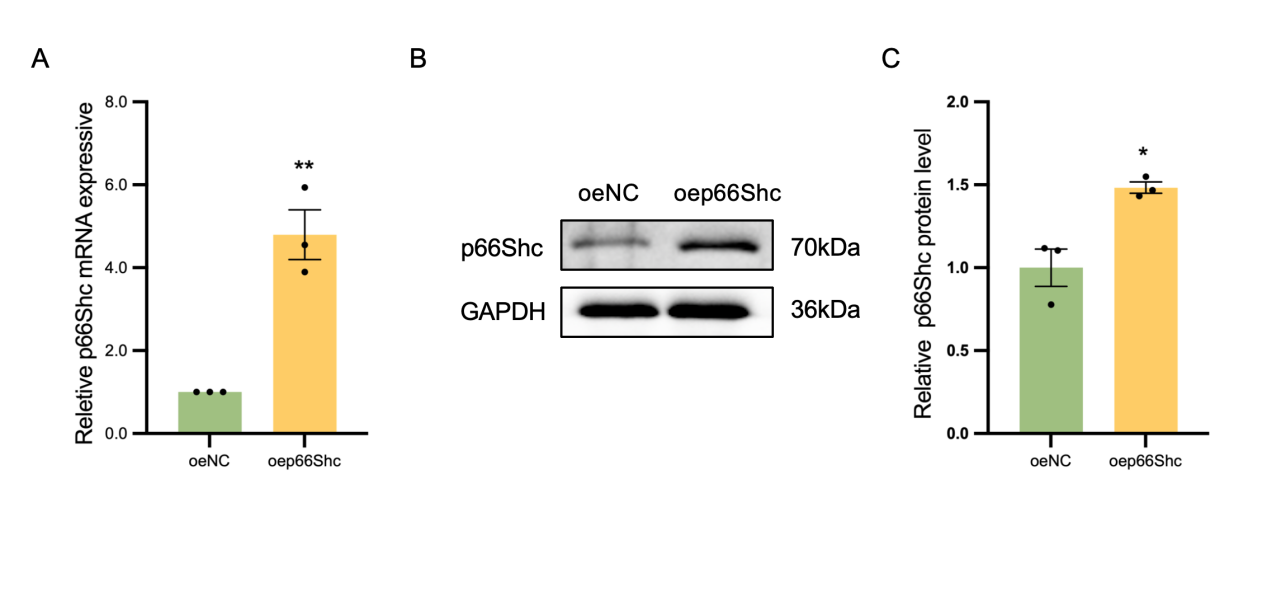


Figure S5. Plasmid transfection efficiency of p66Shc. (A) oep66Shc transfection efficiency was assessed by qPCR. (B) oep66Shc transfection efficiency was assessed by WB. **P* < 0.05; ***P* < 0.01. Data are shown as means ± SEMs (n = 3).


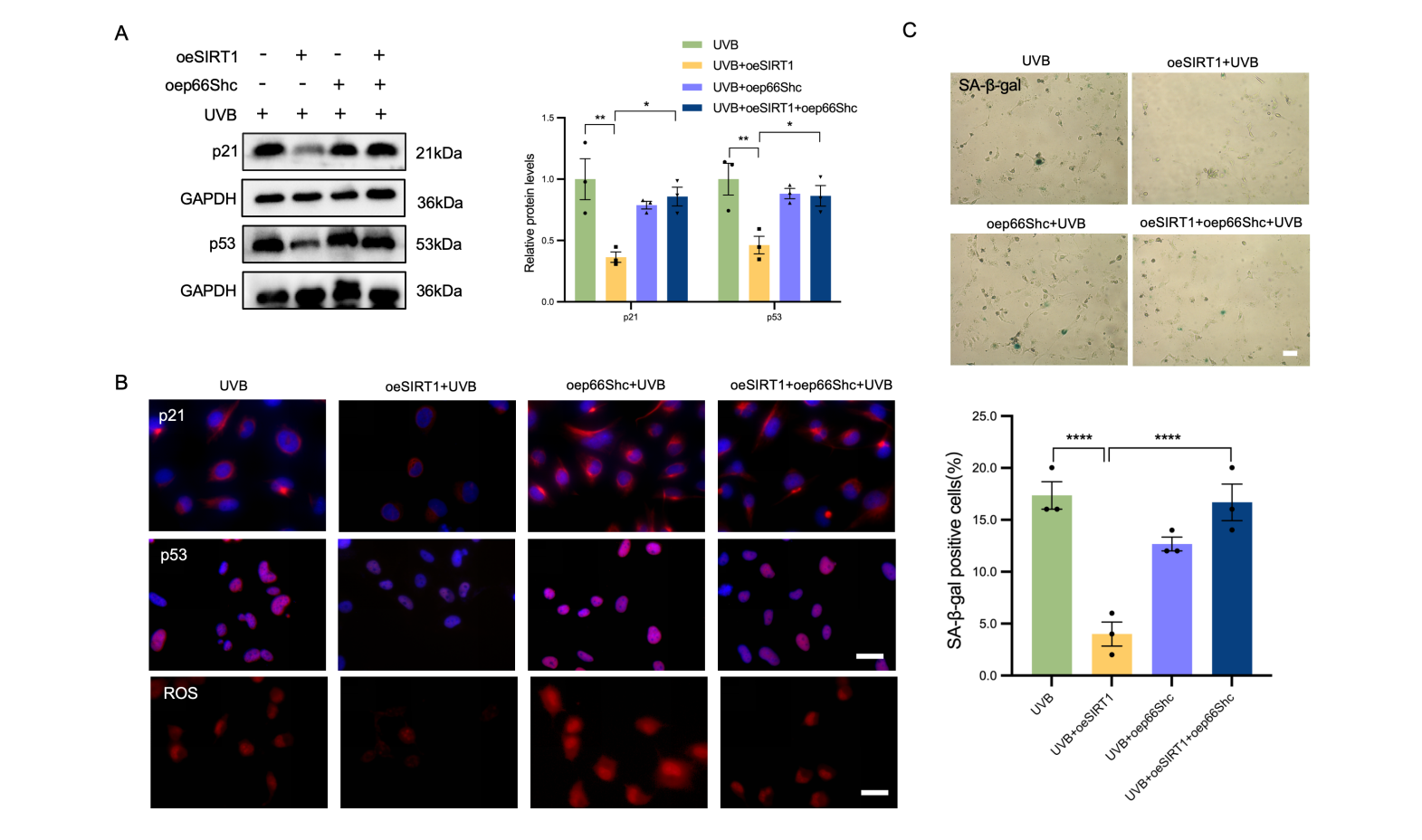


Figure S6. p66Shc overexpression mediated the effects of SIRT1 on UVB-induced LECs senescence. (A) LECs were transfected with the oeSIRT1 plasmid, oep66Shc plasmid or empty vector, and the expression of p21 and p53 was assessed by WB. (B) IF staining and DHE dye of p21 (red), p53 (red) and nuclei (blue) in the UVB, UVB+oeSIRT1, UVB+oep66Shc and UVB+oeSIRT1+oep66Shc groups. Scale bars = 50μm. (C) The percentage of senescent cells was analyzed by SA-β-gal staining. Scale bars = 100μm. **P* < 0.05; ***P* < 0.01; *****P* < 0.0001. Data are shown as means ± SEMs (*n* = 3).


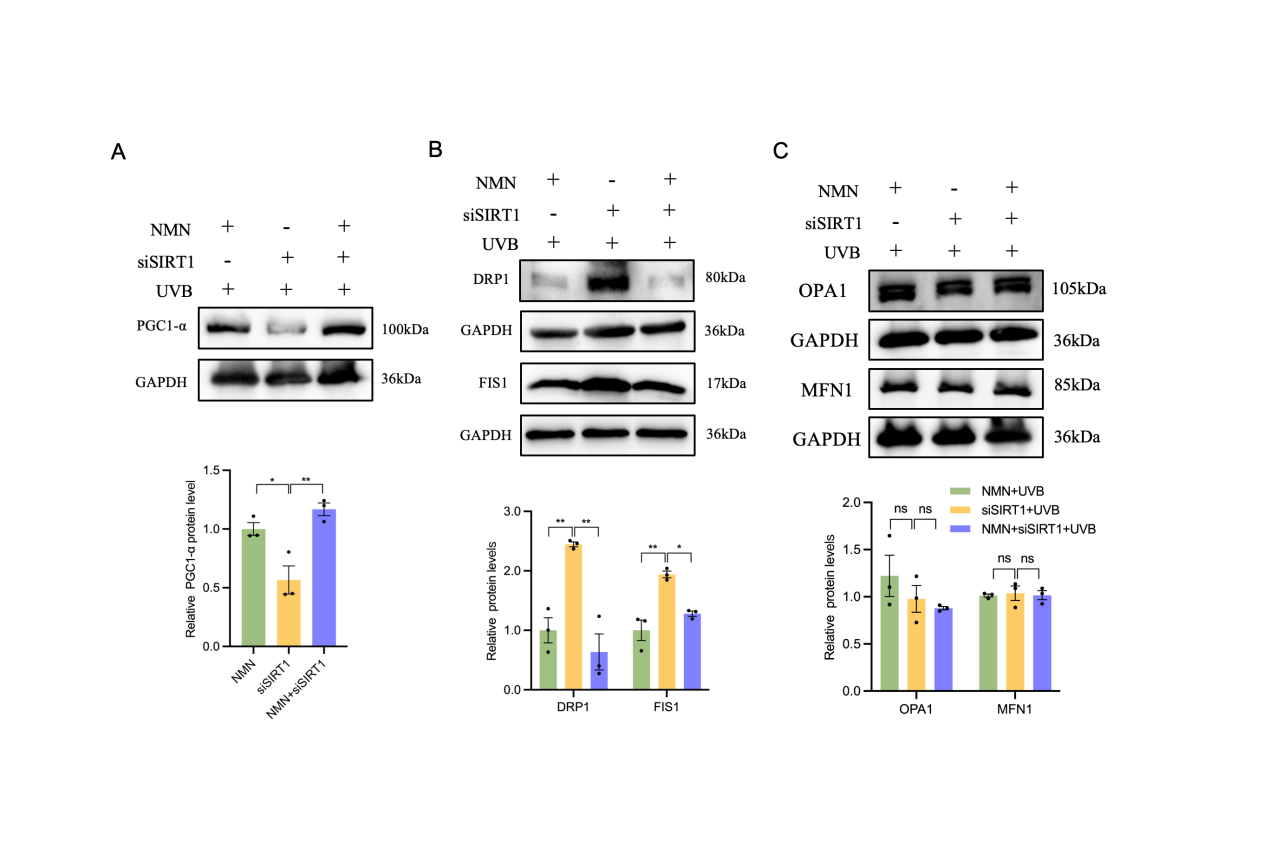


Figure S7. NMN alleviated UVB-induced mitochondrial fission in LECs via SIRT1. (A) Protein levels of the mitochondrial dynamic genes PGC1-α was assessed by WB.(B) Protein levels of the mitochondrial dynamic genes DRP1 and FIS1 were assessed by WB.(C) Protein levels of the mitochondrial dynamic genes OPA1, and MFN1 were assessed by WB. **P* < 0.05; ***P* < 0.01. Data are shown as means ± SEMs (*n* = 3).

Figure S8. The lenses of SD rats were observed under an optical microscope. Scale bar = 1mm.


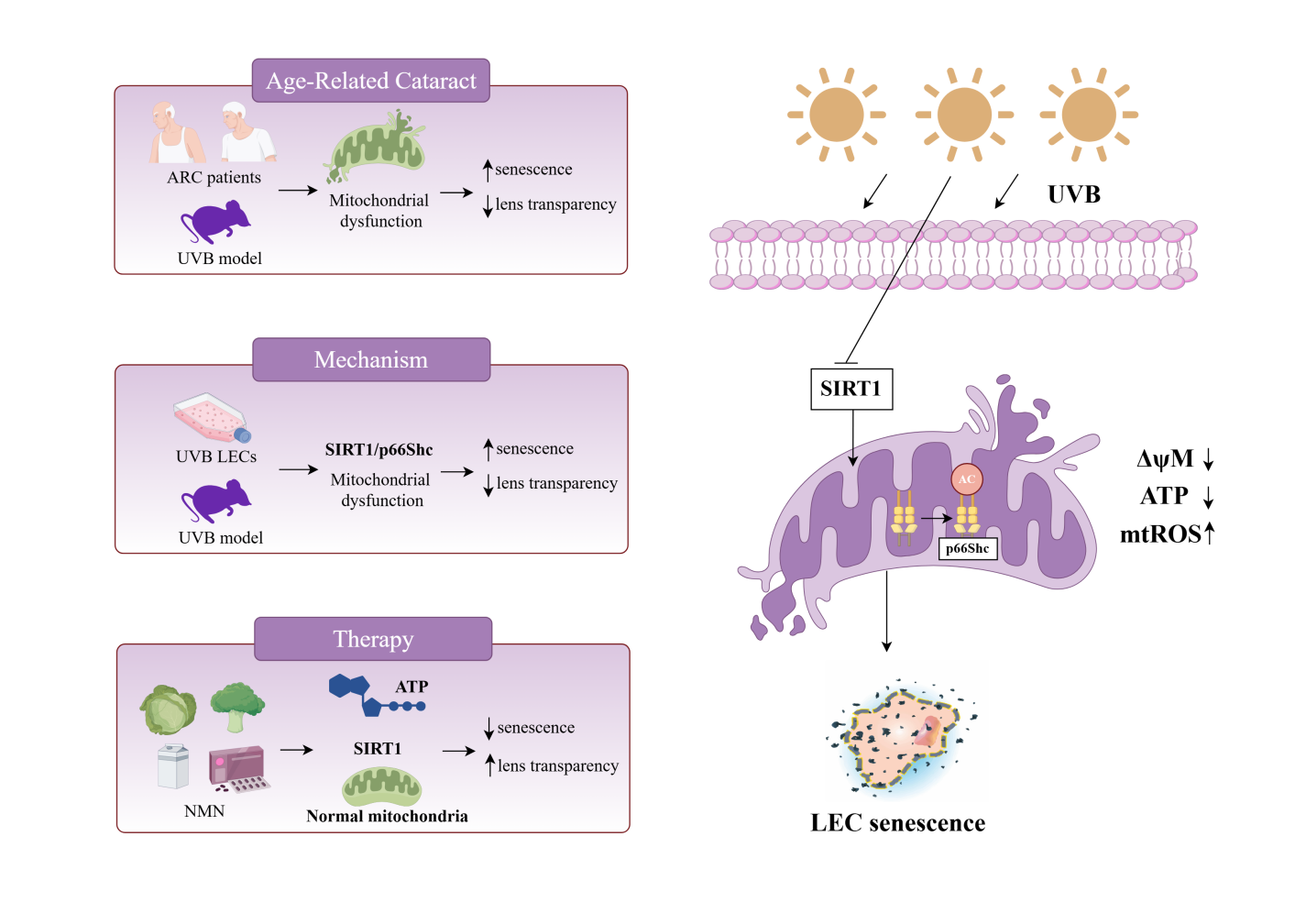
Figure S9. Graphic abstract of the study.
